# A review on microbe–mineral transformations and their impact on plant growth

**DOI:** 10.3389/fmicb.2025.1549022

**Published:** 2025-07-31

**Authors:** Nikita Pradhan, Shikha Singh, Garima Saxena, Nischal Pradhan, Monika Koul, Amit C. Kharkwal, Riyaz Sayyed

**Affiliations:** ^1^Amity Institute of Microbial Technology, Amity University, Noida, India; ^2^Division of Microbiology, Indian Council of Agricultural Research (ICAR)-Indian Agricultural Research Institute (PUSA), New Delhi, India; ^3^Amity Institute of Biotechnology, Amity University, Noida, India; ^4^Department of Botany, Hansraj College, University of Delhi, New Delhi, India; ^5^Department of Biological Sciences and Chemistry, College of Arts and Sciences, University of Nizwa, Nizwa, Oman

**Keywords:** arbuscular mycorrhizal fungi (AMF), biofertilizers, biogeochemical, bioremediation, heavy metal detoxification, microbial nutrient mobilization, microbial–mineral interactions, mineral transformation

## Abstract

Mineral–microbe interaction is a driving environmental changes, regulating the biogeochemical cycling of elements, and contributing to the formation of ore deposits. Microorganisms are fundamental to mineral transformation processes, exerting a profound influence on biogeochemical cycles and the bioavailability of critical nutrients required for plant growth. In this review, we delve into the various mechanisms by which microbes facilitate mineral dissolution, precipitation, and transformation, with a particular focus on how these processes regulate the availability of both macronutrients and micronutrients in soils. Essential microbial activities such as phosphate solubilization, iron chelation, and sulfur oxidation play a pivotal role in enhancing nutrient uptake in plants, thereby supporting sustainable agricultural practices and reducing dependence on chemical fertilizers. Furthermore, microbial-driven mineral transformations are vital for environmental remediation efforts, as they contribute to the immobilization of toxic metals and the detoxification of contaminated soils. By examining key microbial–mineral interactions—including nitrogen fixation, siderophore production, and metal precipitation—this review underscores the indispensable role of microorganisms in improving soil fertility, fostering plant growth, and bolstering ecosystem resilience. The exploration of these microbial processes reveals significant potential for advancing bioremediation strategies and the development of biofertilizers, offering promising solutions to enhance agricultural productivity and address environmental challenges.

## 1 Introduction

Minerals serve as the fundamental constituents of Earth’s materials, with their physical and chemical properties preserving critical information about the environmental conditions prevailing at the time of their formation. Microorganisms, representing the majority of phylogenetic diversity, have been integral agents in shaping Earth’s biogeochemical processes throughout its geological history. Minerals are important for the plant growth and productivity where microbes act as providers for the various minerals that are essential for plant growth (Vessey, 2003; Dong et al., 2022). Plants rely on a diverse array of nutrients, predominantly taken up as mineral ions from the soil, to support critical physiological functions, including photosynthesis, cellular respiration, and enzymatic activity. In addition to the well-recognized roles of macronutrients such as nitrogen (N), phosphorus (P), and potassium (K) in plant nutrition, a suite of micronutrients—such as zinc (Zn), copper (Cu), iron (Fe), and manganese (Mn)—is essential in trace amounts for various enzymatic and metabolic processes (Jabborova et al., 2021a). However, in most soils, these nutrients are often sequestered within insoluble mineral complexes, rendering them largely unavailable for direct plant uptake (Bhat et al., 2024). Therefore, microbes play an important role in the Earth’s Critical Zone (CZ), as they possess a variety of properties that affects dissolution, transformation and formation of minerals through metabolic activities (Lepot, 2020). The process of mineral solubilization in soils is primarily mediated by microorganisms, which are essential in converting insoluble mineral forms into bioavailable nutrients for plant uptake (Kapadia et al., 2022). This microbially driven mineral transformation occurs through diverse biochemical mechanisms, including the secretion of organic acids, enzymes, and siderophores that chelate or solubilize mineral compounds, facilitating their assimilation by plants (Jabborova et al., 2021b; Kusale et al., 2021; Ribeiro et al., 2020). For example, phosphates are an important macronutrient for energy metabolism, root growth and DNA synthesis, but majorly it is present in insoluble forms bound in soil particles like in alkaline and calcareous soil (hydroxyapatite [Ca_10_(PO_4_)_6_(OH)_2_ and tricalcium phosphate [Ca_3_(PO_4_)_2_]) (Elhaissoufi et al., 2021). Phosphate solubilizing bacteria including species of *Pseudomonas, Bacillus*, and *Aspergillus*, secrete organic acids like gluconic, citric, and oxalic acids. Microorganisms, particularly those that produce siderophores—small, high-affinity iron-chelating molecules—play a pivotal role in the mobilization of these essential micronutrients (Nithyapriya et al., 2024). *Pseudomonas, Burkholderia*, and *Streptomyces*, along with the fungi *Trichoderma pseudokoningii* and *Trichoderma longibrachiatum*, produce siderophores that bind to iron ions, sequestering them from insoluble oxide forms. This process increases the solubility of iron, thereby enhancing its availability for plant uptake (Pecoraro et al., 2021). This microbial capability to transform not only macronutrients but also trace elements is fundamental to their role in improving soil fertility and promoting plant growth (Pecoraro et al., 2021).

The widespread use of fertilizers and pesticides has been shown to adversely affect global food production by disrupting the natural composition, structure, and diversity of soil microflora, ultimately compromising soil quality and nutrient availability (Prashar and Shah, 2016; Mandal et al., 2020). For instance, excessive application of nitrogen-based fertilizers leads to soil acidification, while chemical fungicides and insecticides have detrimental effects on soil health (Bitew and Alemayehu, 2017). Enhancing the population of microorganisms that facilitate nutrient transformation in soils can help preserve soil (Wei et al., 2024; Basu et al., 2021). Agricultural practices that promote the proliferation of beneficial microorganisms offer a sustainable, environmentally friendly, and cost-effective alternative to the reliance on agrochemicals and fertilizers, thereby improving soil quality (Eswaran et al., 2024; Wei et al., 2024).

The importance of microbe–mineral interactions in enhancing soil fertility, nutrient availability, and sustainable agriculture has been highlighted in recent research. However, most current literature concentrates broadly on the general contribution microbes play in plant growth, with limited research review to the specific biochemical strategies employed to mobilize or transform mineral-bound nutrients (Alzate Zuluaga et al., 2024). Additionally, little is studied about how these mechanisms function in various soil types and environmental conditions, making a huge research area to be studied and investigated.

In this review, we aim to understand how microbe–mineral interaction plays a pivotal role in environment and agricultural area. The review emphasis on different mechanisms involved in microbe mediated mineral transformation and impact on plant growth and development, underlying the different environmental changes and the soil type. We also have highlighted and summarized the applications and challenges for microbe–mineral interactions in sustainable agriculture and environmental remediation.

## 2 Microbial-mediated mineral transformation processes

Microbial-mediated mineral transformation involves the dynamic interaction between microorganisms and minerals, playing a crucial role in element cycling and driving biogeochemical processes (Ortiz-Castillo et al., 2021). This interaction allows for various applications in geological, environmental, and biotechnological domains. Through various mechanisms such as dissolution, precipitation, and transformation, which in turn impact metal mobility and alter oxidation states (Dong and Lu, 2012). It involves diverse microorganisms which includes prokaryotes and eukaryotes, and their symbiotic associations with each other and higher organisms (Ortiz-Castillo et al., 2021), playing a key role in various applications such as in mine waste management, where microbial communities affect both the formation of secondary minerals and the release of contaminants (Ortiz-Castillo et al., 2021). For example, remediation of chromium and uranium contamination, with microbes transforming aqueous metal ions into amorphous or crystalline precipitates (Cheng et al., 2012). Herein, we elucidate the various types of microbe-mediated processes that occur in nature, along with their underlying mechanisms. These processes include key phenomena such as calcification, silicification, and iron mineralization, which are carried out by a wide array of microorganisms like bacteria, fungi, algae, and viruses.

### 2.1 Mineral dissolution

Mineral dissolution is a key process in which minerals undergo chemical breakdown, typically involving the interaction between a mineral’s solid phase and an aqueous solution. This process plays a significant role in geochemical cycles, environmental remediation, and the availability of essential nutrients (Dong and Lu, 2012). This process can be significantly influenced through the use of microbes that utilize minerals for nutrients or energy, often accelerating the breakdown through biochemical reactions.

In microbial-mediated dissolution, microbes release organic acids or chelating compounds that facilitate the dissolution of minerals by weakening their chemical bonds. For example, *Pseudomonas* strains are capable of extracting zinc, manganese, and aluminum from low-grade ores and secondary minerals through bioleaching processes. Likewise, *Burkholderia* sp. SX9 can mobilize copper, zinc, and cadmium from insoluble oxides or carbonate forms. Siderophores are low-molecular-weight organic compounds produced by microorganisms and plants under iron-limited conditions (Ahmed and Holmström, 2014). These chelators have high affinity for iron and can enhance its solubility from minerals, making it bioavailable (Williamson et al., 2021). Furthermore, it can promote the biodegradation of organic contaminants through reactive oxygen species (ROS) production (Roskova et al., 2022), which is further supported with a study on chrysotile asbestos, where both fungal and bacterial siderophores were shown to remove structural iron from fibers, potentially reducing their toxicity and health risks (Mohanty et al., 2018). Additionally, redox reactions driven by microbial respiration can alter the oxidation states of metals, influencing the solubility of minerals containing those metals. For instance, iron-reducing bacteria can reduce ferric iron (Fe^3+^) to its more soluble ferrous form (Fe^2+^) thus promoting the dissolution of iron-containing minerals. The transformation of elements between different valence states provides a critical energy source for the metabolic systems of many microorganisms. These redox reactions involve the transfer of electrons, releasing varying amounts of energy based on the specific reactants, products, and the metabolic pathways utilized by the microorganisms (Dong and Lu, 2012).

These processes are essential in natural biogeochemical cycles and have both beneficial and detrimental consequences in human contexts, such as bioremediation of metallic and radionuclide pollutants and the development of novel biomaterials.

### 2.2 Mineral precipitation

Mineral precipitation is a critical process in the transformation and formation of minerals, which plays a vital role in bioremediation. This process is driven by the activities of microbes that create local chemical conditions conducive to mineral precipitation. Specifically, microbes can influence the precipitation of metal-bearing minerals by altering redox states, producing reactive metabolites, or creating local environments that favor nucleation and precipitation of minerals (Dong et al., 2022). Mineral precipitation by microorganisms can occur through biologically induced mineralization (BIM), where microbes indirectly modify their local microenvironment leading to extracellular mineral deposition. This happens without direct control over the crystallization process by the microorganisms. BIM typically results from microbial metabolic activities such as metal reduction, oxidation, or the production of metabolic byproducts such as sulfides, oxalates, and carbonates (Han et al., 2020). Biologically Controlled Mineralization (BCM) refers to the process where organisms regulate and direct the formation of minerals. This differs from BIM where external environmental factors trigger mineral precipitation. In BCM, organisms exert precise control over mineral formation, both in terms of location and composition. This process is crucial for the development of specific structures in certain microorganisms, such as the formation of magnetosomes in magnetotactic bacteria, which produce magnetic minerals like magnetite (Fe_3_O_4_) (Hoffmann et al., 2021).

One prominent example is sulfate-reducing bacteria (SRB) that generate sulfide ions as a metabolic byproduct. These ions can react with metal ions such as iron or cadmium, leading to the formation of metal sulfide minerals, which are typically insoluble. This process is essential in environments like contaminated sediments, where SRB can immobilize toxic metals, reducing their bioavailability and mobility, with studies having shown that SRB can remove over 98% of various metal ions from solution, primarily through precipitation and biosorption mechanisms (Neria-González and Aguilar-López, 2021).

Microbial-mediated mineral precipitation is especially relevant in the context of bioremediation. In polluted environments, microbes can transform soluble toxic metals into insoluble mineral forms, effectively removing them from water and soil. For instance, the microbial reduction of soluble uranium U^6+^ to insoluble U^4+^ results in the precipitation of uraninite, a process utilized in the bioremediation of uranium-contaminated groundwater (You et al., 2021). Additionally, microorganisms can promote the precipitation of other environmentally relevant metals like chromium, selenium, and iron, further contributing to bioremediation efforts by immobilizing these elements (Ojuederie et al., 2017).

### 2.3 Microbial transformations

#### 2.3.1 Nitrogen fixation

Nitrogen a crucial nutrient for ecosystems, exists in various forms such as ammonium (NH_4_^+^), nitrate (NO_3_^–^), and nitrogen gas (N_2_). The availability of nitrogen in soils is largely controlled by microbial activity, with bacteria and fungi playing central roles in its transformation, immobilization, and release (Grzyb et al., 2021). These microbial processes are vital for maintaining soil fertility and ensuring that plants receive the mineral nitrogen they need for growth. One of the most important microbial processes affecting nitrogen is nitrogen fixation. Certain bacteria, such as Rhizobium, form symbiotic relationships with legume plants, converting atmospheric nitrogen (N_2_) into ammonia (NH_3_), which plants can readily use (Abd-Alla et al., 2023). This process supplies a significant portion of the nitrogen in many ecosystems. Additionally, free-living nitrogen fixers, such as *Azotobacter* and *cyanobacteria*, contribute to nitrogen fixation in environments without legumes, including in aquatic systems (Lawal, 2021; Rana et al., 2023; Khumairah et al., 2022).

Nitrification is another key microbial process in which bacteria like *Nitrosomonas* and *Nitrobacter* oxidize ammonia into nitrite (NO_2_^–^), and subsequently into nitrate (NO_3_^–^), a form of nitrogen that plants can easily absorb. This process occurs in aerobic soil conditions and is critical for converting ammonia, derived from organic matter or fertilizers, into plant-available nitrates (Lu et al., 2021). However, in anaerobic conditions, a different set of microbes performs denitrification. Bacteria such as *Pseudomonas* and *Clostridium* reduce nitrate back into nitrogen gas (N_2_) or nitrous oxide (N_2_O), thereby removing nitrogen from the soil and releasing it into the atmosphere (Torres et al., 2016). While this process prevents the buildup of nitrates in the environment, it also results in nitrogen loss, impacting soil fertility.

Microbial activity also drives ammonification, the process by which organic nitrogen compounds, like proteins and nucleic acids, are broken down into ammonium (NH_4_^+^) (Lu et al., 2021). Fungi and bacteria decompose complex organic matter, releasing ammonium that can either be taken up directly by plants or further transformed by nitrifying bacteria. Through these interconnected processes—nitrogen fixation, nitrification, denitrification, and ammonification—microbial activity plays a fundamental role in regulating the availability of nitrogen in mineral forms, which is essential for plant growth and the maintenance of healthy ecosystems (Giordano et al., 2021).

#### 2.3.2 Sulfur oxidation

Sulfur, a key mineral nutrient, undergoes various transformations in the soil primarily through microbial activity, which significantly influences its availability to plants and its role in ecosystems. Sulfur exists in different oxidation states, from sulfide (S^2–^) to sulfate (SO_4_^2–^), with microbial processes driving the cycling of sulfur between these forms, impacting its mineralization and bioavailability (Jørgensen et al., 2019).

One of the most critical processes is sulfate reduction, carried out by SRB in anaerobic environments. These microorganisms use sulfate as a terminal electron acceptor, reducing it to hydrogen sulfide (H_2_S). This biogenic sulfide can then react with metal ions to form metal sulfides like pyrite (FeS_2_), contributing to mineral transformations in sediments (Neria-González and Aguilar-López, 2021).

This process is crucial in environments such as wetlands and marine sediments, where oxygen is limited.

Conversely, sulfur oxidation is carried out by sulfur-oxidizing bacteria, such as *Acidithiobacillus ferrooxidans* and *Thiobacillus neapolitanus*. These bacteria oxidize sulfide minerals or elemental sulfur to sulfate, which is a more plant-available form of sulfur (Wang et al., 2019). This microbial oxidation is particularly important in soils with high sulfide concentrations, such as those exposed to mining activities, where it can lead to the formation of acid mine drainage—a detrimental environmental issue.

Microorganisms also facilitate sulfur mineralization through the decomposition of sulfur-containing organic matter. In this process, fungi and bacteria break down organic sulfur compounds, releasing inorganic sulfate that can be assimilated by plants. This microbial activity plays a pivotal role in nutrient cycling, as it ensures the continuous supply of sulfur in its mineral form to support plant growth (Narayan et al., 2023).

#### 2.3.3 Phosphorus solubilization

Phosphorus is a vital macronutrient essential for several biological processes in plants, such as photosynthesis, energy transfer (through ATP), nucleic acid synthesis, and membrane production (Wang et al., 2025). However, most phosphorus in the soil is not readily available to plants, as it is often present in insoluble forms. Soil microorganisms, including bacteria, fungi, and mycorrhizal fungi, play a significant role in the solubilization and mobilization of phosphorus, enhancing its availability to plants (Chen et al., 2023; Pan and Cai, 2023).

Microorganisms contribute to phosphorus solubilization through several biochemical mechanisms. Phosphate-solubilizing bacteria (PSB) like, *Pseudomonas* sp., *Rhizobium* sp., and *Escherichia* sp. and fungi *Talaromyces albobiverticillius* HNB9, release organic acids such as acetic acid, citric acid, and gluconic acid, which lower the pH and dissolve insoluble phosphate complexes in the soil. This process converts phosphorus into a form that can be absorbed by plant roots as orthophosphate (Lee et al., 2012; Kharkwal et al., 2024). Additionally, enzymes like phosphatases, phytases, and C-P lyases produced by these microorganisms mineralize organic phosphorus compounds, making them accessible to plants (Ipsilantis et al., 2018).

For instance, arbuscular mycorrhizal fungi (AMF) form symbiotic relationships with plant roots and significantly aid in phosphorus uptake. The extraradical hyphae of AMF extend beyond the depletion zones around plant roots, exploring larger soil volumes for phosphorus acquisition. These hyphae secrete phosphatases that hydrolyze organic phosphorus and release orthophosphate. In exchange, plants provide carbohydrates to the fungi (Das et al., 2022).

Another example of microbial phosphorus solubilization can be observed in leguminous plants like mung beans (*Vigna radiata*). When coupled with PSB, such as those from the genera *Serratia* and *Enterobacter*, along with inorganic phosphorus fertilizers, these bacteria enhance phosphorus acquisition and promote nitrogen fixation in the plant’s root nodules. This dual action increases phosphorus and nitrogen availability, boosting overall plant growth and yield (Das et al., 2022).

#### 2.3.4 Iron sequestration

Microbial roles in iron transformation play a crucial role in the microbial-mediated mineral transformation of iron, essential for many metabolic activities within cells, including electron transfer during respiration and photosynthesis. Iron is abundant in the environment, but it often exists in forms like hydroxides and oxides, which are not easily accessible to plants due to low solubility (Kermeur et al., 2023; Sayyed et al., 2012).

Microorganisms aid in iron solubilization by producing siderophores, which are small, high-affinity iron-chelating compounds. These siderophores bind to ferric iron (Fe^3+^), transforming it into a soluble form that can be taken up by both plants and microbes (Bridge and Johnson, 2000). For example, the fluorescent *Pseudomonas* species produces siderophores that enhance the iron-acquisition capacity of plants. The iron-siderophore complex is then transported into microbial or plant cells through specific membrane receptors (Gomes et al., 2024). These bacteria can also synthesize other siderophores like pyochelin, pseudopaline, and thioquinolobactin, which may have additional functions such as antimicrobial activity (Timofeeva et al., 2022).

Moreover, the microbial transformation of iron can result in the formation of specific iron minerals. For instance, the bacterium *Geobacter sulfurreducens* reduces Fe^3+^ to Fe^2+^, which can then react with carbonate to form minerals like siderite (FeCO_3_). Additionally, the reduction of Fe^3+^ by bacteria often leads to the formation of magnetite (Fe_3_SO_4_), a mixed-valence iron oxide, which is important in both environmental and geological contexts (Han et al., 2020).

## 3 Effects of mineral solubilization on plant growth and development

### 3.1 Nutrient uptake and assimilation

Plant–microbe interactions significantly influence plant growth, development, and resilience to environmental challenges. These interactions span nutrient acquisition, stress alleviation, and their broader applications in agriculture (Etesami and Adl, 2020). This section discusses the direct mechanisms of microbial-mediated nutrient uptake, the indirect strategies for stress mitigation, and specific applications for enhancing agricultural productivity.

#### 3.1.1 Direct mechanisms of nutrient uptake

Microbial communities associated with plant roots play critical roles in facilitating the uptake of essential nutrients (Table 1). These mechanisms are crucial in nutrient-deficient environments, contributing to the sustainability of agricultural systems by reducing dependency on chemical fertilizers.

**TABLE 1:** Microbial mechanisms enhancing plant macro and micronutrient uptake (Bhat et al., 2024).

Nutrient	Microbial mechanism	Key microorganisms	Effect on plant growth
Nitrogen (N)	Nitrogen fixation	*Rhizobium, Azotobacter, Nostoc*	Convert atmospheric nitrogen into ammonium for plant use
Phosphorus (P)	Phosphate solubilization	*Bacillus, Rhizobium, Enterobacter*	Converts insoluble phosphorus into soluble forms, enhancing growth
Potassium (K)	Potassium solubilization	*Clitopilus hobsonii, Aspergillus aculeatus*	Releases K from minerals, promoting growth under K-limited conditions
Iron (Fe)	Siderophore production	*Rhizobium, Pseudomonas*	Chelates Fe^3+^ into bioavailable Fe^2+^ for plant uptake
Zinc (Zn)	Zinc mobilization	*Pseudomonas, Bacillus*	Enhances Zn uptake, boosts photosynthesis, and gene expression for Zn tolerance
Manganese (Mn)	Mn solubilization	*Aspergillus terreus, Acinetobacter*	Improves Mn availability, critical for stress tolerance in low-Mn soils

##### 3.1.1.1 Nitrogen fixation

Nitrogen is a vital macronutrient for plants, serving as a building block for proteins, nucleic acids, and chlorophyll. However, plants cannot directly utilize atmospheric nitrogen (N_2_). Nitrogen-fixing microbes, such as *Rhizobium*, *Frankia*, *Azotobacter*, and *cyanobacteria* (Nostoc), convert N_2_ into ammonium (NH_4_^+^) through the enzymatic activity of nitrogenase, a complex enzyme requiring ATP and low oxygen conditions for functionality (Talaat and Shawky, 2014; Ramesh et al., 2023).

Symbiotic bacteria like *Rhizobium* establish nodules on leguminous roots, forming mutualistic associations where plants provide carbon while bacteria fix nitrogen. Non-symbiotic nitrogen fixers, such as *Azospirillum*, colonize the rhizosphere and enhance nitrogen availability for cereals and non-leguminous crops (Glick, 2012). These interactions contribute to significant reductions in nitrogen fertilizer application, promoting sustainable agriculture (Normand et al., 2007). Studies have shown that inoculating soybeans with *Bradyrhizobium japonicum* raised grain protein content by 28% and improved yield by 15% through enhanced nitrogen absorption and biomass growth. In a multi-location field study in Northern Ghana, using commercial strains like Biofix dramatically increased nodule numbers (up to 118%), dry weight (more than twofold), and grain yield (12%–19%) compared to uninoculated controls, showcasing substantial symbiotic efficiency and economic viability (Ulzen et al., 2016).

##### 3.1.1.2 Phosphate solubilization

Phosphorus is essential for energy transfer, photosynthesis, and genetic material synthesis in plants. Despite its abundance in soil, phosphorus is often locked in insoluble forms, such as calcium phosphate or aluminum phosphate complexes. Phosphate-solubilizing microbes (PSM), including *Bacillus*, *Pseudomonas*, and *Penicillium*, produce organic acids like citric, oxalic, and gluconic acids, which dissolve these complexes into plant-available forms (monobasic and dibasic phosphate ions) (Miliute et al., 2015). These microbes also release phosphatases that hydrolyze organic phosphorus compounds, further enhancing soil fertility. For example, inoculating wheat and maize with PSB has demonstrated increased phosphorus uptake, root biomass, and grain yield (Mitter et al., 2013; Rawat et al., 2021). A recent study indicated that *Pseudomonas putida* enhanced phosphorus acquisition efficiency in maize by 33% and increased dry biomass by 19%, underscoring the significance of PSM in nutrient uptake and growth (Singh et al., 2024).

##### 3.1.1.3 Potassium mobilization

Potassium is critical for enzymatic activation, photosynthesis, and osmoregulation in plants. However, most soil potassium is bound in feldspar, mica, and other silicate minerals, making it unavailable for plant uptake (Baba et al., 2021). Potassium-solubilizing bacteria (KSB), such as *Acinetobacter* and fungi like *Aspergillus aculeatus*, employ acidolysis, chelation, and other biochemical processes to release potassium ions from these minerals (Sardans and Peñuelas, 2021; Peng et al., 2021). Field trials have shown that inoculating potassium-deficient soils with KSB enhances potassium availability, resulting in improved crop performance, particularly under stress conditions. For instance, perennial ryegrass inoculated with *Aspergillus* exhibited better potassium uptake, photosynthesis, and biomass production (Sardans and Peñuelas, 2021). Additionally, *Bacillus mucilaginosus* enhanced potassium uptake and grain yield by 25% in wheat, emphasizing its importance in plant growth and nutrient uptake under potassium stress. Similarly, under salt-affected soil conditions, the combination of *Bacillus circulans* inoculation with 1. Notably, 5% K-leaf foliar application significantly improved the physiological and biochemical traits of wheat, resulting in a 16. 41% increase in grain yield and enhanced potassium uptake, while decreasing the harmful Na^+^/K^+^ ratio (El-Egami et al., 2024).

##### 3.1.1.4 Iron chelation

Iron, though abundant in the earth’s crust, is often inaccessible to plants due to its tendency to oxidize into insoluble ferric form (Fe^3+^). Plant growth-promoting rhizobacteria (PGPR), such as *Pseudomonas fluorescens* and *Rhizobium*, produce siderophores—iron-chelating molecules that bind Fe^3+^ and reduce it to bioavailable ferrous form (Fe^2+^) (Ahemad and Kibret, 2014; Sayyed et al., 2012). This process not only enhances iron uptake but also reduces pathogen proliferation by depriving them of iron. Siderophore-producing microbes have been particularly effective in managing iron deficiencies in crops like wheat and rice while promoting plant vigor under iron-limited conditions. For instance, siderophore-producing Rhizobium increased chlorophyll content by 35% and shoot dry weight by 20% in rice grown in iron-deficient soils, revealing the relationship between microbial iron chelation and improved growth performance (Ahemad and Kibret, 2014).

##### 3.1.1.5 Micronutrient solubilization (Zn and Mn)

Micronutrients such as zinc (Zn), and manganese (Mn) play vital roles in enzymatic activity, photosynthesis, and stress tolerance. Zinc-deficient soils are common globally, necessitating external supplementation. Zn-mobilizing bacteria like *Bacillus* and *Pseudomonas* solubilize zinc compounds through organic acid production, siderophore secretion, and rhizosphere acidification (Kamran et al., 2017). Recent findings by Kamran et al. (2017) showed that various rhizobacterial strains, including *Pseudomonas fragi*, *Pantoea dispersa*, and *Enterobacter cloacae*, significantly enhanced both shoot and root dry weights, as well as zinc content in wheat tissues. Notably, *P. fragi* improved zinc translocation to grains, indicating that zinc-solubilizing rhizobacteria not only promote biomass growth but also enhance zinc bioavailability and uptake efficiency in wheat under zinc-deficient conditions.

In a related study at the National Agriculture Research Center (NARC), Islamabad, during 2020–2021, several promising indigenous bacterial strains, such as *Pantoea* sp., *Klebsiella* sp., and *Acinetobacter* sp. were identified for their ability to significantly boost wheat growth and zinc biofortification. Inoculating with these zinc-solubilizing bacteria led to a 14% increase in shoot length and up to 14% enhancement in root dry weight, alongside an impressive 1177% rise in zinc content in wheat shoots compared to the control, highlighting the potential of native bioinoculants for sustainable zinc management (Ali et al., 2023).

Manganese availability is often limited in well-aerated, calcareous soils. Acidophilic bacteria (*Acinetobacter* and *Lysinibacillus*) and fungi (*Aspergillus terreus*) release Mn^2+^ ions, making manganese accessible to plants. These interactions enhance the photosynthetic efficiency and stress tolerance of crops such as soybeans and rice (Nagaraju et al., 2024; Sanket et al., 2017).

#### 3.1.2 Indirect mechanisms: stress alleviation

Microbial communities mitigate abiotic stresses by improving water retention, regulating stress-responsive hormones, and detoxifying harmful substances. These mechanisms are critical for enhancing crop resilience under adverse environmental conditions (Table 2).

**TABLE 2:** Microbial mechanisms for abiotic stress alleviation.

Abiotic stress	Microbial mechanism	Key microorganisms	Impact on plant	Citations
Drought	EPS production, ABA regulation, osmolytes	*Bacillus*, *Azospirillum*	Enhances water retention and drought tolerance	Ahmad et al., 2022; Cohen et al., 2015
Salinity	ACC deaminase, ionic balance, antioxidants	*Pseudomonas*, *Azospirillum*	Reduces ion toxicity and oxidative stress	Ayuso-Calles et al., 2021; Navarro et al., 2014
Heavy metals	Siderophores, biosorption, metal transformation	*Saccharomyces cerevisiae*, *Pseudomonas*	Decreases metal toxicity, improves phytoremediation	Saha et al., 2016; Ullah et al., 2015

##### 3.1.2.1 Drought stress

Drought stress is caused by limited water availability, which severely affects plant growth, development, and productivity, particularly in arid and semi-arid regions. It reduces turgor pressure, stunted growth, wilting, and impaired photosynthesis, which lowers crop yields (Ilyas et al., 2020). Additionally, drought induces oxidative stress and increases ethylene levels, accelerating leaf senescence (Khan et al., 2019). Recent studies have shed light on the vital role that certain drought-tolerant microbes, such as PGPR (like *Bacillus subtilis*) and AMF (such as *Glomus intraradices*), play in helping plants cope with drought conditions. These microorganisms enhance the resilience of plants through various interconnected mechanisms, making a significant difference when water is scarce (Ilyas et al., 2020).

For instance, PGPR like *B. subtilis* and *P. fluorescens* produce substances called exopolysaccharides (EPS), which improve soil structure and increase its ability to hold water. This creates a safer environment for plants, helping them survive during dry spells (Sheteiwy et al., 2021; Ilyas et al., 2020). Additionally, some PGPR have a unique capability to reduce ethylene levels in stressed plants by breaking down the precursor to this gas [1-aminocyclopropane-1-carboxylate (ACC)]. Lower ethylene levels mean that plants can slow down aging and keep their photosynthesis running smoothly even under drought stress (Khan et al., 2021a). These helpful microorganisms also contribute to the production of osmolytes, like proline, glycine betaine, and soluble sugars, within host plants, which assist in maintaining the right balance of water in cells. At the same time, they enhance the activity of vital antioxidant enzymes, such as superoxide dismutase (SOD), catalase (CAT), and peroxidases (POD), which protect plants by detoxifying harmful ROS and ensuring cellular balance during drought conditions (Nader et al., 2024; Wu and Zou, 2017).

**FIGURE 1:**
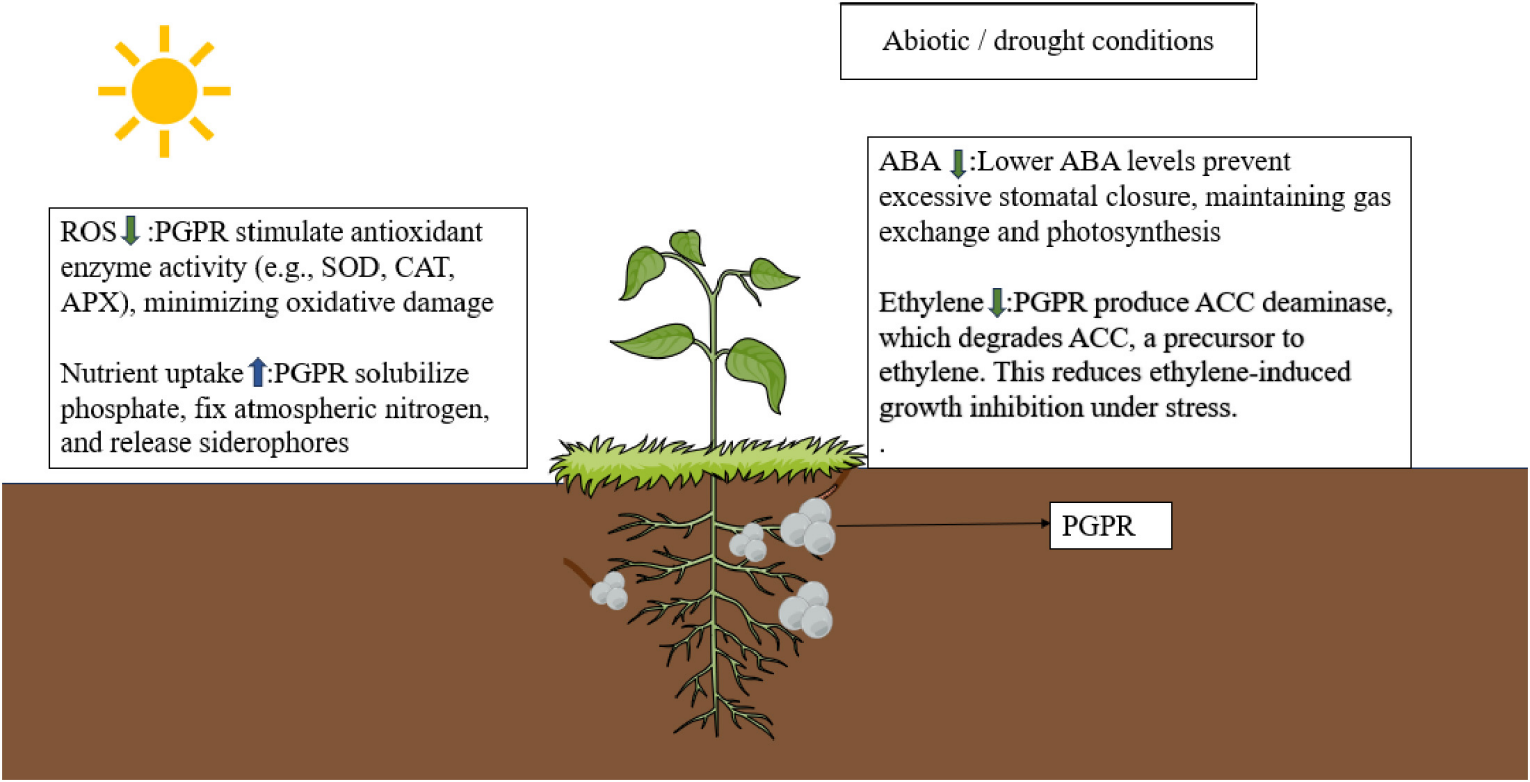
The figure highlights function of plant growth-promoting rhizobacteria (PGPR) in relieving abiotic stress and increasing plant growth in conditions of drought. PGPR improve drought resistance in plants by minimizing oxidative stress through the induction of antioxidant enzymes (SOD, CAT, and APX) and enhancing nutrient acquisition through phosphate solubilization, nitrogen fixation, and siderophore production. PGPR regulate hormone response by inhibiting ABA levels to preserve stomatal function and photosynthesis. PGPR also produce ACC deaminase, which breaks down ACC and decreases ethylene synthesis and ethylene-induced growth inhibition during stress. Figure created using BioRender. Root structure was illustrated in a style inspired by published figures from Dr. Guillaume Lobet (ORCID: 0000-0002-5883-4572).

Another important aspect is how these microbes help regulate plant hormones. Drought-resistant microbes can elevate levels of abscisic acid (ABA), a crucial hormone that helps plants close their stomata and conserve water. They also boost auxins like indole-3-acetic acid (IAA), which encourage roots to grow longer and branch out, allowing plants to absorb more water (Ahmad et al., 2020; Cohen et al., 2015). Moreover, AMF, such as *G. intraradices*, form beneficial partnerships with plant roots, significantly improving how well roots can take up water and nutrients, particularly phosphorus. When these fungi colonize plant roots, they prompt the expression of proteins called aquaporins and increase compatible solutes in plant tissues, further enhancing water retention and tolerance to drought (Sheteiwy et al., 2021) (Figure 1).

##### 3.1.2.2 Salinity stress

Salinity stress, mainly caused by high levels of sodium chloride (NaCl), can be quite detrimental to plants. It leads to osmotic stress, hinders their ability to absorb water, and often results in dehydration and stunted growth (Tiwari et al., 2016). Beyond this, it can cause ion toxicity, disrupt how nutrients are taken up, diminish chlorophyll content, and negatively impact photosynthesis (Al-Turki et al., 2023). Much like drought, salinity stress initiates oxidative stress by producing ROS, which can harm cells and upset nutrient balance, ultimately degrading membrane integrity and cellular function (Tiwari et al., 2016).

A study conducted by Ahmed et al. (2023) found that inoculating maize with *Funneliformis constrictum* can help these plants cope with salinity by enhancing their antioxidant defenses and polyamine metabolism. In light of increasing seawater stress, AMF were found to alleviate oxidative damage by reducing levels of hydrogen peroxide (H_2_O_2_) and malondialdehyde (MDA). They also boosted the activity of key antioxidant enzymes such as CAT, SOD, and ascorbate peroxidase (APX). Additionally, AMF increased amino acid levels, which promoted the production of important polyamines like putrescine, spermidine, and spermine. This was achieved by activating certain biosynthetic genes while suppressing those responsible for breaking down these compounds. These changes significantly improved the plants’ ability to endure stress and support growth under high-salinity conditions.

Moreover, PGPR, which have the ability to break down ACC deaminase, can help stressed plants by lowering ethylene levels. This process aids in preventing premature aging and encourages root growth (Damodaran et al., 2013). These beneficial microorganisms also enhance the activity of antioxidant enzymes like SOD, CAT, and APX, which aid in neutralizing ROS and restoring redox balance. In summary, these microbial partnerships can significantly enhance plant resilience against drought and salinity stresses by improving water retention, modulating hormonal levels, and reducing oxidative damage.

##### 3.1.2.3 Phytoremediation and heavy metal detoxification

Phytoremediation is a green and sustainable approach to cleaning up soil that’s been contaminated with heavy metals such as cadmium (Cd), lead (Pb), arsenic (As), and beryllium (Be). This method becomes even more effective with the help of plant growth-promoting microorganisms (PGPM), which assist in extracting these harmful metals through various processes, including their accumulation inside and outside plant cells, their sequestration, and transforming them into less toxic forms (Ullah et al., 2015; Ma et al., 2016).

Specifically, beneficial strains like *Pseudomonas*, *Bacillus*, and *Azospirillum* play a key role in reducing metal stress on plants. They do this by producing siderophores, which are small molecules that can bind to iron and heavy metals such as Cd, Ni, and Zn, thereby affecting how these metals move and are absorbed by plants (Złoch et al., 2016; Saha et al., 2016). In addition to metal binding, PGPM also releases organic acids, like citric, oxalic, and malic acids, which help to lower the pH of the soil around plant roots, making metal ions more soluble and accessible for uptake (Ullah et al., 2015). They also produce various enzymes, including nitrogenase and phosphatase, which facilitate nutrient cycling and bolster plant health, especially in contaminated environments. Moreover, PGPM helps plants grow by providing essential nutrients, managing pathogens, and improving metal accumulation capacity. When combined with AMF, this partnership significantly enhances the effectiveness of phytoremediation by boosting nutrient absorption and resistance against heavy metals (Vigliotta et al., 2016).

Recent studies have shown that inoculating plants with AMF such as *Rhizophagus irregularis* can help reduce beryllium (Be) toxicity in ryegrass and chickpeas. This leads to decreased Be absorption and improved physiological resilience. When AMF are present, plants display better photosynthetic performance, less oxidative stress (as indicated by lower levels of hydrogen peroxide and lipid damage), and an increase in protective substances like proline and sucrose. These beneficial changes are associated with better nitrogen metabolism and increased activity of an enzyme called nitrate reductase, with ryegrass showing an even stronger response than chickpeas (Sheteiwy et al., 2022).

#### 3.1.3 Specific case studies and applications

##### 3.1.3.1 PGPR in improving crop yield

Inoculation with PGPR like *Azospirillum brasilense* and *B. subtilis* has consistently shown improvements in root architecture, nutrient uptake, and resistance to abiotic stresses. Field applications in crops such as wheat, maize, and rice demonstrate increased yields and reduced fertilizer dependency (Etesami and Glick, 2024).

##### 3.1.3.2 Mycorrhizal fungi in enhancing resource use efficiency

Arbuscular mycorrhizal fungi (*Glomus mosseae*) extend their hyphal networks beyond the root zone, enhancing phosphorus and water uptake in resource-limited environments (Vafa et al., 2021). Their application in crops like barley and maize has shown improved nutrient efficiency and drought tolerance (Ferrol et al., 2016).

### 3.2 Root growth and architecture

Plant–microbe interactions profoundly influence root architecture and rhizosphere dynamics, which significantly impact plant performance and soil ecosystem functions. Root architecture traits, such as branching, elongation, and exudation, are essential for nutrient acquisition and stress adaptation and are heavily influenced by microbes like AMF and PGPR. These microbes enhance root development, modulate exudates, and improve nutrient uptake, particularly under stress conditions. Additionally, rhizodeposition, the release of organic compounds from roots, plays a crucial role in shaping rhizosphere microbial communities by attracting beneficial microbes and supporting nutrient cycling. This section discusses microbial effects on root traits, rhizodeposition processes, and their role in shaping rhizosphere microbial communities (Vafa et al., 2024).

Root phenotypic traits, such as branching, elongation, and surface area, are crucial for nutrient acquisition, stress tolerance, and plant–microbe interactions. Microbes significantly influence these traits, promoting root development and enhancing functionality. Mycorrhizal fungi, especially AMF, extend root systems through fungal hyphae, leading to increased root branching, lateral root formation, and enhanced root length, thereby improving nutrient uptake (Rawat et al., 2021). Similarly, PGPR produce phytohormones like IAA, which stimulate root elongation and branching, increasing the root surface area for nutrient acquisition. For example, inoculation with *P. fluorescens* significantly improved lateral root density in wheat, thereby enhancing phosphate uptake (Etesami and Adl, 2020). Under stress conditions, root plasticity plays a key role in adaptation, such as increased root length and surface area in response to low phosphorus availability. PGPR further augment these adaptive traits by influencing root exudate composition, thereby enhancing microbial colonization and nutrient availability in the rhizosphere (Bouremani et al., 2023). Rhizodeposition, involving the release of organic compounds such as sugars, amino acids, and secondary metabolites from roots, is pivotal in shaping the rhizosphere microbiome. These exudates not only provide carbon sources for soil microbes but also act as chemical signals that attract beneficial microbes, including AMF and PGPR, while repelling pathogens. For instance, maize roots release flavonoids that promote AMF colonization, improving phosphorus uptake and drought tolerance (Zhang et al., 2015). Rhizodeposition is most concentrated at root tips, where microbial colonization is typically highest. Root traits, such as growth angle, rooting depth, and lateral root density, influence the spatial distribution and persistence of exudates, thereby shaping the rhizosphere microbial community. Root system architecture, including root growth angle and depth, also determines microbial community composition. Shallow roots are associated with processes like nitrification and organic matter degradation, whereas deeper roots facilitate reductive processes such as denitrification (Galindo-Castañeda et al., 2022). Moreover, axial and lateral root traits significantly affect microbial interactions; plants with more axial roots enhance microbial colonization through increased carbon deposition via exudates, while higher lateral root branching density provides additional attachment points for microbes, fostering diverse microbial communities and improving nutrient uptake under phosphorus-limited conditions (Chen and Liu, 2024). Collectively, these microbe-mediated interactions influence root architecture and exudation patterns, enhancing nutrient cycling, stress resilience, and soil health. Such interactions underscore the potential for sustainable agricultural practices and reveal strategies for improving crop productivity, particularly in resource-limited environments (Shah et al., 2021) (Table 3).

**TABLE 3:** Microbial applications in agriculture.

Application	Microbial group	Mechanism	Example crops	Citations
Biofertilizers	*Rhizobium*, AMF, *Bacillus*	Nitrogen fixation, phosphate solubilization	Legumes, maize, wheat	Mitter et al., 2013; Etesami and Glick, 2024
Climate change mitigation	Cyanobacteria, *Sporosarcina*	Carbon fixation, biomineralization	All agricultural crops	Mitchell et al., 2010; Khan et al., 2021b
Bioremediation	*Pseudomonas*, *Saccharomyces*	Hydrocarbon degradation, heavy metal sequestration	Contaminated agricultural soils	Ullah et al., 2015; Saha et al., 2016

### 3.3 Plant stress tolerance

Abiotic stresses such as drought, salinity, and heavy metal toxicity limit crop yield by disrupting metabolic and physiological processes including water uptake, ion homeostasis, photosynthesis, and oxidative balance. Beneficial microbes such as PGPR, AMF mitigate these effects through multiple synergistic mechanisms (Table 2).

Microorganisms play a vital role in enhancing plant resilience under challenging environmental conditions. During drought stress, for instance, many soil microbes secrete exopolysaccharides (EPS), which help bind soil particles together, improving both soil structure and its capacity to retain water. This not only facilitates better root access to moisture but also leads to the formation of a protective biofilm around the rhizosphere (Ahmad et al., 2020). PGPR equipped with the enzyme ACC deaminase further assist in managing drought stress by breaking down ACC, a precursor to ethylene, a hormone that tends to accumulate during stress and accelerates plant senescence. By lowering ethylene levels, these microbes support prolonged root growth and delayed aging of plant tissues (Sagar et al., 2020; Ayuso-Calles et al., 2021). In parallel, beneficial microbes stimulate the accumulation of osmoprotective compounds such as proline, glycine betaine, and soluble sugars. These molecules help maintain cellular integrity and enzyme function during osmotic stress by balancing the internal water potential (Eswaran et al., 2024). Microbial activity also influences plant hormone levels, particularly increasing ABA, which regulates stomatal closure to conserve water, and auxins, which promote root elongation and branching, traits critical for improved water uptake under limited moisture conditions (Cohen et al., 2015; Tanveer et al., 2023). AMF, like *G. intraradices*, add another layer of drought defense by enhancing root hydraulic conductivity and upregulating aquaporin genes, thus boosting water transport efficiency in plants (Sheteiwy et al., 2021).

Under salinity stress, microbes help mitigate ionic imbalance and toxicity. One key strategy is enhancing the selective absorption of potassium (K^+^) over sodium (Na^+^), preserving ionic homeostasis. At the same time, microbial partners activate the plant’s antioxidant machinery, elevating enzymes such as SOD, catalase (CAT), and APX, which detoxify ROS and protect plant cells from oxidative damage (Hamidian et al., 2023; Kapadia et al., 2022; Gupta et al., 2013). For example, *P. putida* has been shown to improve salinity tolerance in rice by enhancing both antioxidant activity and ion regulation mechanisms. Interestingly, ACC deaminase activity again plays a dual role here, limiting ethylene accumulation under salinity stress, thereby supporting continued root development (Sagar et al., 2020; Ayuso-Calles et al., 2021). Certain AMF species, like *F. constrictum*, contribute by modulating polyamine pathways, increasing compounds such as putrescine and spermidine. These not only stabilize cell membranes but also help maintain ionic balance within plant tissues (Ahmed et al., 2023).

When it comes to heavy metal stress, microbes aid plants in multiple ways. Some bacteria, notably those that produce siderophores like *Pseudomonas* and *Bacillus*, bind toxic metals such as cadmium (Cd) and nickel (Ni), thereby reducing their availability and toxicity. Others secrete organic acids that chelate metals, improving their uptake by roots for eventual detoxification (Złoch et al., 2016; Saha et al., 2016; Sagar et al., 2022). AMF like *R. irregularis* limit the movement of heavy metals into the shoots, thereby protecting photosynthetic tissues. They also boost the plant’s antioxidant system and support better photosynthetic efficiency under stress (Sheteiwy et al., 2022; Ullah et al., 2015). Altogether, the collaboration between PGPR and AMF forms a multi-tiered defense mechanism, biochemical, physiological, and molecular that significantly enhances plant adaptation and tolerance to a variety of abiotic stressors.

#### 3.3.1 Biotic stress tolerance

Soil and plant roots host a variety of pathogens and beneficial microbes. Biotic stress caused by pathogens can lead to imbalanced hormonal regulation, nutrient deficiencies, and physiological disorders (Ramegowda and Senthil-Kumar, 2015). Using PGPM as a biological control agent offers a sustainable alternative to chemical pesticides. PGPM promotes plant growth and development by enhancing nutrient uptake, activating defense mechanisms, and suppressing pathogens. Co-inoculation with PGPR and mycorrhizae can improve plant resistance to biotic stress (Alkilayh et al., 2024).

##### 3.3.1.1 Mechanism of biotic stress tolerance

Plant–microbe interactions are crucial for managing biotic stress. PGPM can induce systemic resistance (ISR) and systemic acquired resistance (SAR) in plants. Non-pathogenic root-associated microbes activate ISR, while SAR involves changes in gene expression and the production of defense-related proteins. PGPM can also produce allelopathic compounds, siderophores, and antibiotics that inhibit pathogen growth. For instance, *Bacillus* strains have effectively managed diseases like bacterial leaf blight and black rot through ISR (Ghazalibiglar et al., 2016; Vinothini et al., 2024; Anbalagan et al., 2024; Paliwal et al., 2023). Microbial solubilization of minerals (MSMs) plays a crucial role in promoting plant growth and development by improving nutrient availability and activating defense mechanisms, particularly against biotic stressors such as pathogens and pests. A prominent defense strategy supported by MSMs is ISR, a comprehensive immune response that primes plants to respond swiftly and effectively to biotic challenges. ISR operates through signaling pathways regulated by jasmonic acid (JA) and ethylene (ET), which coordinate proactive defense strategies (Ali et al., 2022). The JA pathway begins with the synthesis of jasmonic acid from linolenic acid in the chloroplast, followed by its conversion to jasmonoyl-isoleucine (JA-Ile), the bioactive form. JA-Ile binds to the COI1 receptor, triggering the degradation of JAZ repressors and activating transcription factors like MYC2. These transcription factors drive the expression of defense-related genes, enhancing the production of pathogenesis-related (PR) proteins, antimicrobial secondary metabolites, and enzymes that reinforce cell walls. Simultaneously, the ET pathway, derived from methionine through the Yang cycle, stabilizes EIN3, a key transcription factor, upon ethylene binding. This pathway complements JA signaling, enabling a robust and comprehensive defense, particularly effective against necrotrophic pathogens (Ruan et al., 2019).

Beyond triggering ISR through microbe-associated molecular patterns (MAMPs), MSMs enhance plant resilience by solubilizing essential nutrients such as phosphorus, iron, and potassium. For instance, microbial siderophores chelate iron, supporting the production of ROS, which serve as an early line of defense against pathogen invasion. Solubilized calcium and silicon further strengthen plant cell walls, providing a physical barrier against infection (Tiwari et al., 2024). Additionally, ISR stimulates the synthesis of secondary metabolites like flavonoids and phytoalexins, which act as potent antimicrobial compounds. A notable advantage of ISR is its ability to balance growth and defense by conserving resources until an actual threat is encountered. This ensures plants maintain their growth and development even under stress conditions. Moreover, ISR offers broad-spectrum resistance, reducing disease severity and improving overall plant health (Pusztahelyi et al., 2015). The use of ISR-inducing MSMs as bioinoculants in agriculture presents an eco-friendly and sustainable strategy to enhance crop resilience and productivity while minimizing dependence on chemical pesticides. By combining nutrient solubilization with immune activation, MSMs contribute significantly to sustainable agricultural practices and stress management in crop production systems.

##### 3.3.1.2 Hormonal modulation

Plant growth-promoting rhizobacteria can influence plant hormonal levels, promoting root and shoot development, which enhances overall plant growth and productivity:

Auxin production: IAA is a widely studied auxin involved in plant growth and development. It is primarily produced in plant buds and young leaves through various biosynthetic pathways (Kundan et al., 2015). IAA promotes key processes such as cell division, differentiation, elongation, and wall extensibility in young stems, improving the plant’s ability to absorb water and nutrients. It also regulates apical dominance, bud formation, and adventitious and lateral root development (Grobelak et al., 2015). Additionally, IAA plays a role in leaf and flower abscission. Other auxin-like compounds include indole-3-acetamide, indole-3-pyruvate, and 4-chloroindole-3-acetic acid (Olanrewaju et al., 2017).
Gibberellin production: GAs regulate key growth processes, including seed germination, stem elongation, flowering, and fruit setting. They enhance photosynthesis and chlorophyll content, promote shoot growth, and inhibit root growth through GA signaling and DELLA repressors (Martínez et al., 2016).
Cytokinin production: cytokinins produced by some PGPR can delay plant aging (senescence) and promote cell division, leading to improved plant vigor and yield.
Ethylene inhibition: ACC deaminase, produced by some PGPR, promotes plant growth by lowering ethylene levels, a hormone linked to stress responses in plants. Excess ethylene, triggered by biotic and abiotic stresses, negatively affects plant shoot and root development (Gamalero and Glick, 2015). ACC deaminase reduces ethylene by converting its precursor, ACC, into α-ketobutyrate and ammonia, thereby restoring normal plant growth (Olanrewaju et al., 2017). PGPR application helps plants manage stress by reducing ethylene-induced damage.

## 4 Microbial–plant–mineral interactions

Plants need minerals both macronutrients and micronutrients for their growth and form a symbiotic association with microbes for their uptake from soil. This interaction between plants, microbes and minerals is a key system for the biosphere. Their interaction with microbes increases plant growth and overall health, crop productivity, mineral uptake, plant defense, soil fertility, etc. Through improved soil fertility and weathering of rocks, vital nutrients (N, P, and K) can be acquired from minerals (Ahemad and Kibret, 2014). Various plant–microbial associations such as mycorrhizal, cyanobacterial, nitrogen fixers, etc. contribute to plant nutrition by increasing the soil health and mineral cycles. Microbial activity supports the breakdown of organic matter by degradation, dissolution, transformation, and precipitation which releases crucial minerals back to the soil while minerals provide protection to microbes from harsh environments such as physical abrasion, thermal fluctuation and UV irradiation (Dong et al., 2022). Understanding these interactions and their mechanisms are very essential for sustainable agriculture and ecosystem management.

### 4.1 Rhizosphere processes

**FIGURE 2:**
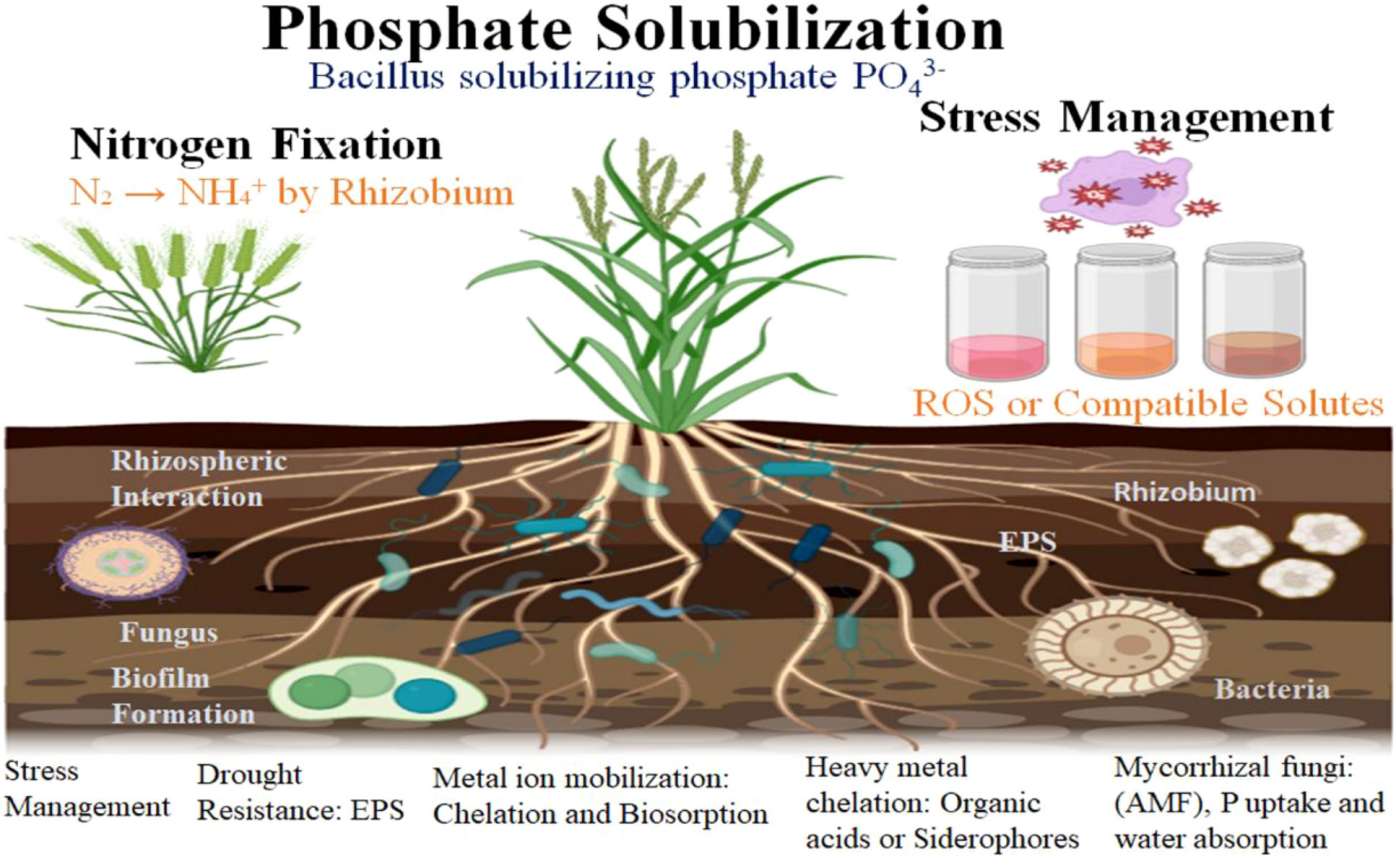
The figure highlights the interactions between plants and microbes in the rhizosphere, showcasing processes such as nitrogen fixation (Rhizobium converting N_2_ to NH_4_^+^), phosphorus solubilization by microbes like *Bacillus* and *Pseudomonas*, and stress adaptation mechanisms like EPS production for drought tolerance and heavy metal detoxification by fungi such as *Aspergillus*. These microbial activities boost nutrient uptake, support plant growth, and enhance stress resistance. The figure has been made using Bio render.

Rhizosphere is the key area where microbial–plant–mineral interactions occur. In rhizosphere, soil is directly influenced by plant roots and microbial activities. Plant roots exude low molecular weight organic compounds which aggressively support microbial colonization. In natural ecosystem, most mineral nutrients are in their non-available forms and their availability rely on soil pH, organic acids, microbial activity, etc (Zaidi et al., 2015). Microbes such as plant growth promoting rhizobacteria, diazotrophs, AMF, and cyanobacteria are well known for mineralization process through solubilization, absorption, oxidization, fixation, etc (Figure 2). Microorganisms maintain soil structure by improving aeration and water retention which helps in plant growth. In natural environment, these microbial mediated mineral transformations are rate-limiting and essential processes for plant growth, and ecosystem abundance (Thepbandit and Athinuwat, 2024). So, understanding the dynamics of the rhizosphere is requisite for enhanced agricultural practices in future.

### 4.2 Mycorrhizal associations

Arbuscular mycorrhizal fungi represent an obligate symbiotic relation with plant roots which colonize the 80% of terrestrial plants including cereals, vegetables, legumes, fruits, medicinal plants, and various commercial crops such as sunflower, sugarcane, cotton, etc. (Saboor et al., 2021; Jabborova et al., 2022; Vafa et al., 2024). AMF promote the uptake and transfer of solubilized minerals from soil toward the plant roots through extra radical mycelium (ERM) which absorbs water and nutrients beyond the depletion zones and in-turn gets the photosynthates (carbohydrates) from the plants. AMF spores infect the root cortical cells, travel by hyphae inside the root and form arbuscule which is the key component for nutrient exchange (Liu et al., 2022). AMF hyphae network can lengthen up to 25 cm around the plant root and help in establishment of other microbes as well by enhancing the root zone 10%–100% (Smith and Read, 2008). Various studies showed under drought stress where the mobility of P and N is low, AMF can effectively uptake the nutrients through ERM. By dipeptide transporters, *R. irregularis* efficiently assimilate organic N from soil likewise *G. mosseae* membrane Pi transporters (PT) govern the Pi repositioning from soil to ERH (Khoso et al., 2025).

### 4.3 Plant growth-promoting rhizobacteria

Plant growth-promoting rhizobacteria are useful symbiotic bacteria which make a niche in rhizosphere and strengthen the plant growth by several mechanisms such as absorption of nitrogen and phosphorus, phytohormone production, mineral solubilization, iron chelating siderophore production, etc. (Parray et al., 2023). The bacterial potential to solubilize mineral P increase the availability of phosphate and iron for plants. They solubilize insoluble P by the production of organic acids- acetic acid, butyric acid, citric acid, lactic acid, oxalic acid, maleic acid etc. *Pseudomonas, Bacillus*, and *Rhizobium* are some common bacteria which participate in P solubilization. Similarly, reduction in pH is also reported by *Staphylococcus saprophyticus* SM7 and *Staphylococcus haemolyticus* MS7 when solubilize the mineral Zn in medium (Javaid et al., 2023). By the process of biological nitrogen fixation (BNF) microbes can fix atmospheric nitrogen and make it available for plant use. Significant nitrogen-fixers such as *Rhizobium, Azospirillum, Azotobacter*, etc. are used for growing cash crops and some other cereals now a days along with leguminous crops (Basu et al., 2021). Some of the efficient mineral solubilizing bacteria (MSB) are from the genera *Bacillus licheniformis, Arthrobacter, Enterobacter, Bacillus cereus, Staphylococcus, B. subtilis, Paenibacillus polymyxa, Bacillus aryabhattai* (Javaid et al., 2023; Basu et al., 2021; Ahmad et al., 2020; Thomas et al., 2018). So, MSB provide an encouraging replacement to the usage of traditional chemical fertilizers, because they augment the bioavailability of mineral nutrients in soil which enhance plant growth, development, defense and hence crop productivity and yield. The application of such MSB strains in crops can overcome mineral deficiency in plants which can be environmentally and profitably more noteworthy in future.

## 5 Impact of microbial mineral transformation on plant growth

**FIGURE 3:**
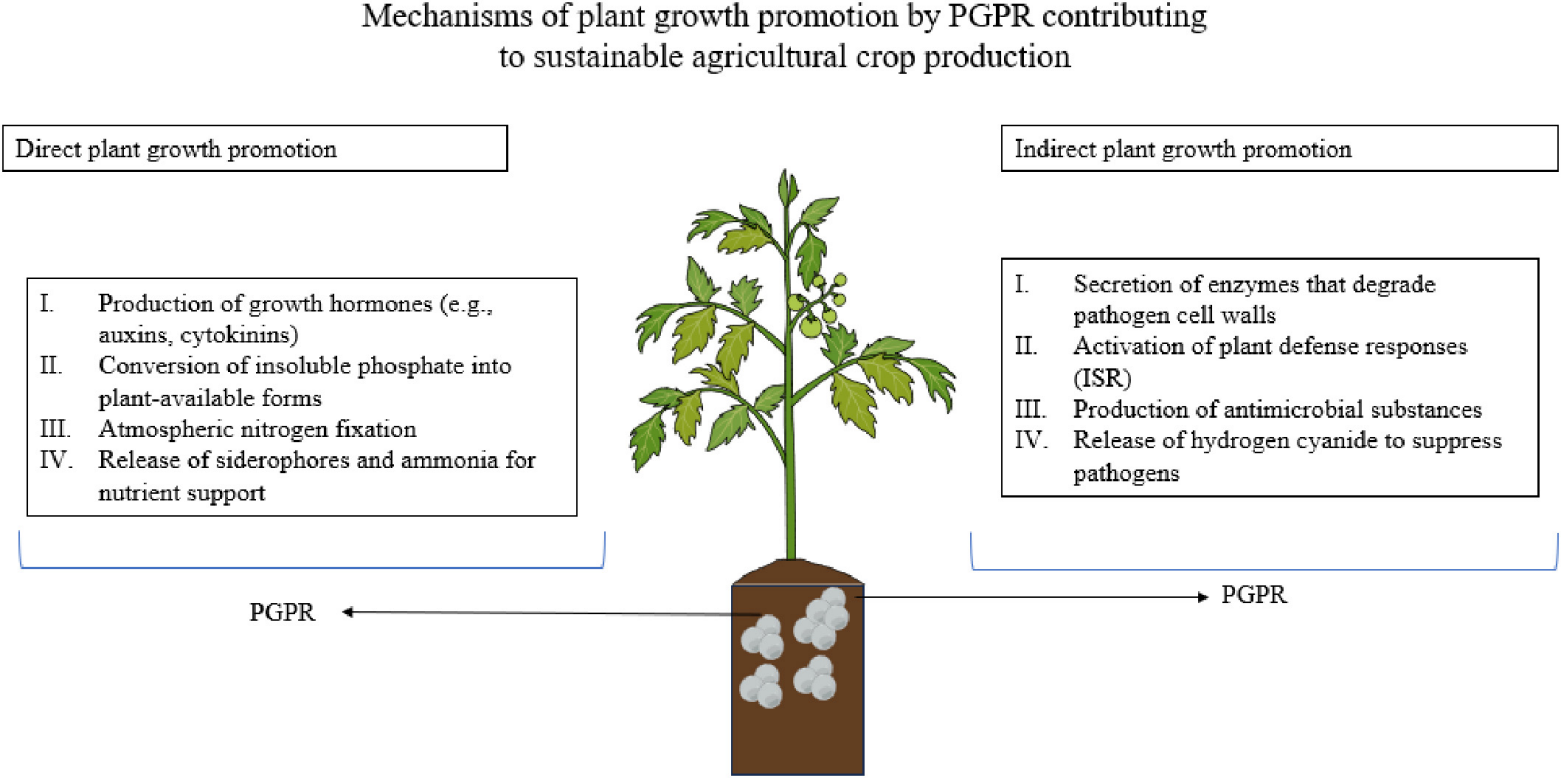
The figure highlights the mechanisms through which plant growth-promoting rhizobacteria (PGPR) increase crop yield are by both direct and indirect means. This figure demonstrates the two-way function of PGPR in sustainable agriculture. Direct mechanisms are production of phytohormones (e.g., auxins and cytokinins), solubilization of phosphate, nitrogen fixation, and siderophore and ammonia production for improving nutrient availability. Indirect mechanisms are induction of plant defense through production of cell wall-degrading enzymes, induction of induced systemic resistance (ISR), production of antimicrobial compounds, and production of hydrogen cyanide to inhibit phytopathogens. Figure created using BioRender. Root structure was illustrated in a style inspired by published figures from Dr. Guillaume Lobet (ORCID: 0000-0002-5883-4572).

Microbial transformations play a critical role in influencing the availability of both macro and micronutrients to plants, directly impacting soil fertility and crop productivity (Figure 3). These transformations primarily occur within the rhizosphere, where the interactions between plant roots and beneficial microbes help regulate nutrient cycling, solubilization, mobilization, and translocation processes.

Microorganisms, such as PGPR, fungi, and cyanobacteria, enhance the availability of nutrients through a series of biochemical processes. These include the production of organic acids, enzymes, and other metabolites that convert unavailable forms of nutrients into ones that plants can absorb more efficiently (Singh et al., 2020).

One of the most important microbial processes for nutrient availability is solubilization. Microbes enhance the solubilization of essential elements such as phosphorus, zinc, and potassium by secreting organic acids, which lower soil pH and help dissolve minerals bound in insoluble forms (Paul and Parihar, 2021). For example, specific species of *Bacillus* and *Pseudomonas* produce citric, oxalic, and malic acids, which play a significant role in solubilizing zinc and iron, making them more accessible to plant roots (Da Silva et al., 2023; Kuhad et al., 2011). These microbial actions are especially important in soils where these nutrients tend to precipitate and become unavailable to plants under normal conditions.

Mobilization is another key microbial function that enhances nutrient availability. Certain microbes release chelating compounds, such as siderophores, that bind to metals like Fe, Mn, and Zn, aiding their transport into plant systems (Singh et al., 2020). This microbial activity is particularly beneficial in alkaline or calcareous soils, where nutrient availability is often limited due to the formation of insoluble complexes. By mobilizing these micronutrients, microbes ensure a steady supply to the plants, thus supporting their metabolic functions and overall growth (Vélez-Bermúdez and Schmidt, 2022).

Redox reactions mediated by microbes also significantly affect nutrient availability, particularly for elements like iron and manganese. In soils with low oxygen levels, microbes convert oxidized forms of these nutrients, such as Fe^3+^ and Mn^4+^, into their reduced, more soluble states (Fe^2+^ and Mn^2+^), which are readily absorbed by plant roots (Jones et al., 2020). These biological reduction processes are essential for maintaining nutrient supply in flooded or poorly drained soils, where the availability of such micronutrients would otherwise be limited.

In addition to these chemical transformations, microbes can alter the physical structure of plant roots to enhance nutrient uptake (Saeed et al., 2021).

For example, PGPR play a crucial role in enhancing plant growth and development through various mechanisms. They colonize the rhizosphere and modify root system architecture by producing phytohormones and other signals, leading to increased lateral root branching and root hair development (Vocciante et al., 2022). PGPR improve nutrient uptake efficiency, tolerance to abiotic stressors, and crop quality through direct and indirect mechanisms (Rouphael and Colla, 2018). Moreover, these modifications in root architecture are often accompanied by an increase in the density and efficiency of nutrient transporters in the root cells, further improving nutrient acquisition.

Through these various mechanisms, microbial transformations significantly improve the availability of essential nutrients to plants. This not only leads to enhanced plant growth and higher crop yields but also contributes to better nutritional quality in food crops, as plants more efficiently accumulate important minerals such as zinc, iron, and manganese.

Recent developments in microbe–mineral interactions have greatly expanded our understanding of their diverse roles in enhancing plant health and supporting sustainable agriculture. Over the past 10 years, research has increasingly recognized the crucial influence of PGPR especially PSB in boosting soil fertility and crop productivity. These beneficial microbes not only aid in nutrient mobilization but also contribute to a range of plant-supportive functions, including improved tolerance to environmental stresses. Field experiments consistently demonstrate that microbial interactions with soil minerals can increase the availability of essential nutrients such as phosphorus, potassium, and micronutrients, often translating into significant yield improvements. In soils that are degraded or contaminated, integrated approaches using customized biochar in combination with PSB or PGPR–plant systems have achieved over 60% immobilization of toxic metals while simultaneously restoring vital soil health indicators (Zhang et al., 2025).

Within the broader PGPR category, PSB have emerged as effective biofertilizers capable of enhancing nutrient uptake, stimulating plant growth, and raising overall crop yields. Recent studies have also shown that PSB can mitigate abiotic stresses such as drought and salinity by modulating plant hormone pathways and activating defense mechanisms (Al-Turki et al., 2023). Long-term field studies further confirm their effectiveness, particularly in crops like maize and peanuts. For example, peanut yields have increased by up to 19.5% when PSB are applied alongside other beneficial microorganisms (Wang D. et al., 2024). These treatments have also been associated with improved nitrogen use efficiency and elevated seed protein levels, highlighting their contribution to crop quality.

Similarly, KSB have shown promise in enhancing crop performance while reducing reliance on chemical fertilizers. In wheat, KSB application has been linked to increases in root length ranging from 16% to 40% and biomass gains of two- to threefold compared to untreated controls (Gandhi et al., 2022). In multi-year trials conducted in India, cold-tolerant *Pseudomonas* strains (L3 + P2) led to a 22% rise in grain yield and a 16% increase in total biomass relative to uninoculated plots (Dasila et al., 2023). Under dryland farming conditions, co-application with phosphorus fertilizers has resulted in yield gains of up to 63% (Peng et al., 2024).

Beyond nutrient dynamics, certain microbial species such as *B. subtilis* MP1 have demonstrated the ability to accelerate silicate mineral weathering and enhance the accumulation of inorganic carbon in soils. Field data indicate sequestration rates of up to 2.02 tonnes of carbon per hectare per year, accompanied by increased levels of important soil cations like Ca^2+^, Mg^2+^, and Fe^2+^ (Timmermann et al., 2025). Altogether, these findings underscore the practical benefits of leveraging microbe–mineral strategies to boost soil fertility, promote carbon storage, and rehabilitate degraded ecosystems—offering a pathway toward more resilient and environmentally sustainable agricultural systems.

### 5.1 Soil structure and fertility: the role of microbial mineral transformation in soil aggregation

Soil structure is intricately linked to fertility, with soil aggregates playing a pivotal role in the retention of nutrients, water, and organic matter. Aggregates, composed of mineral particles and organic carbon, are held together by electrostatic interactions and encrusted organic materials, forming microaggregates (<250 μm) and macroaggregates (0.25–2 mm) (Wilpiszeski et al., 2019). These structures influence soil fertility by controlling water retention, nutrient flow, and microbial habitat availability. Microbial processes are central to the formation and stabilization of these aggregates, especially through microbial mineral transformations. Soil microorganisms, particularly bacteria and fungi, contribute to aggregate stability by producing extracellular polymeric substances (EPS), which bind soil particles together. This biotic activity not only enhances the mechanical strength of aggregates but also plays a significant role in geochemical cycling (Costa et al., 2018). Moreover, microbes also participate in the transformation of soil minerals. For example, microbial activity can lead to the destruction of rock-forming minerals and the formation of new minerals, such as calcite and iron-containing compounds (Maksimovich et al., 2021).

Microorganisms also play a crucial role in shaping porosity of soil through their interactions with organic matter and minerals. The architecture of soil aggregates, shaped by microbial activity, has a direct effect on nutrient availability and soil fertility. Pore spaces created by microbial transformations facilitate the movement of water and nutrients, while also providing protection to microbial communities within the aggregates. Microorganisms living in these confined spaces help maintain the balance between oxygen and nutrient levels, contributing to essential processes like nitrogen fixation and denitrification.

Furthermore, microbial decomposition of organic matter leads to the formation of water-stable macro-aggregates, with microbial-derived carbon acting as a cementing agent (Rabbi et al., 2019). The resulting pore networks within aggregates vary in size and connectivity, influencing the transport of soluble organic carbon, nutrients, and contaminants (Guhra et al., 2022). Pore size distribution affects microbial community composition and associations, with larger pores promoting diversity under low hydraulic connectivity (Xia et al., 2021). These microbial-driven changes in soil architecture directly impact carbon decomposition rates and bacterial diversity. The complex interplay between substrate availability, microbial activity, and pore geometry creates a dynamic system that influences soil function and carbon cycling.

Additionally, microbial by-products such as organic acids and enzymes also facilitate the weathering of minerals, contributing to soil aggregation and the formation of organ mineral complexes that further stabilize the soil matrix.

### 5.2 Specific example of microbe–plant interactions and their outcomes on plant growth

#### 5.2.1 Case study: mycorrhizal fungi and rock weathering

A notable example of specific microbe–plant interactions and their outcomes on plant growth occurs with mycorrhizal fungi in forest ecosystems. Mycorrhizal fungi form symbiotic relationships with plant roots, where they provide plants with critical nutrients in exchange for carbohydrates (Drijber and McPherson, 2021). In nutrient-poor soils, particularly in boreal forests, these fungi secrete organic acids that dissolve minerals like feldspar and apatite, releasing phosphorus and calcium into the soil (Luo et al., 2024). This weathering process not only sustains forest fertility but also plays a crucial role in global nutrient cycles.

In agricultural systems, this process has been harnessed to enhance crop productivity in areas with poor soil fertility. For example, PSB are known to release phosphate from insoluble mineral sources such as apatite through the secretion of organic acids (Pang et al., 2024). This microbial action provides a natural and sustainable alternative to synthetic phosphate fertilizers, improving soil fertility while reducing the environmental impact of agriculture.

#### 5.2.2 Case study: biofertilizers

Another practical application of microbial mineral weathering is the use of biofertilizers containing weathering microbes. In India, farmers in regions with low-phosphorus soils have used biofertilizers enriched with PSB to improve crop yields. A meta-analysis revealed an average yield improvement of 1.59 tonnes per hectare due to biofertilizer application, with the highest effects observed in cereals (Praveen and Singh, 2019). PSB, often combined with AMF, can significantly enhance plant growth, nutrient uptake, and crop yield, as demonstrated in wheat cultivation in Rajasthan (Saxena et al., 2014). The effectiveness of PSB as biofertilizers varies based on their solubilization mechanisms and molecular composition, highlighting the need for further research to identify efficient strains for agricultural use.

#### 5.2.3 Case study: common bean and plant growth-promoting bacteria

In a study involving common beans inoculated with *Bacillus* sp., *Pseudomonas* sp., *Serratia* sp., *Trichoderma koningiopsis*, and *Burkholderia* sp., significant improvements in plant growth and yield were observed. These microorganisms enhanced nutrient uptake, especially nitrogen, through mechanisms such as phosphate solubilization and BNF (Jalal et al., 2024). The presence of these bacteria facilitated increased root biomass, leading to more efficient water and nutrient absorption. For example, P*seudomonas* sp. is efficient PSM that produce organic acids, primarily gluconic acid, to solubilize inorganic phosphates (Blanco-Vargas et al., 2020).

#### 5.2.4 Case study: wheat and zinc-solubilizing bacteria

Wheat has been effectively biofortified with zinc through the inoculation of zinc-solubilizing bacteria such as *B. subtilis* and *Acinetobacter* sp. These bacteria release organic acids that solubilize zinc from insoluble soil compounds, making it bioavailable to the plant (Yadav et al., 2023). Inoculation with these microbes not only improved zinc uptake but also enhanced wheat growth, productivity, and grain zinc concentration.

#### 5.2.5 Maize and diazotrophic bacteria

Maize plants inoculated with diazotrophic bacteria such as *A. brasilense* and *Burkholderia cepacia* displayed improved growth and nitrogen content in the leaves. These bacteria fix atmospheric nitrogen and make it available to the plant, which reduces dependency on nitrogen fertilizers (Kumar et al., 2022). Furthermore, *Azospirillum* produces phytohormones like IAA, which stimulate root development, enhancing water and nutrient uptake (Jalal et al., 2024).

## 6 Applications in agriculture and biotechnology

Advances in plant–microbe interactions have paved the way for eco-friendly solutions to enhance crop productivity and address environmental challenges. These applications, including biofertilizers, climate change mitigation, sustainable agriculture, and bioremediation, underscore the role of beneficial microbes in modern agricultural systems (Table 3).

### 6.1 Biofertilizers: enhancing nutrient availability

Biofertilizers are substances containing living microorganisms that enhance plant growth by improving the availability of essential nutrients like nitrogen, phosphate, and iron when applied to seeds, plant surfaces, or soil. Nitrogen-fixing bacteria such as *Rhizobium* and *Azotobacter* play a crucial role in biofertilizers. These bacteria reside in the nodules of plant roots, particularly in legumes, and transform atmospheric nitrogen into organic forms that plants can utilize (Talaat and Shawky, 2014; Alami et al., 2024). Similarly, cyanobacteria convert environmental nitrogen into ammonia for plant uptake. Phosphate is another vital nutrient for plants, crucial for stress tolerance, maturity, and quality. *Penicillium bilaii*, a beneficial fungus, enhances phosphate availability by producing organic acids that dissolve soil phosphates, making them accessible to plant roots (Mosttafiz et al., 2012; Miliute et al., 2015; Rawat et al., 2021).

Iron, though abundant in nature, is often unavailable to plants in its ferric form (Fe^3+^). PGPRs address this by secreting siderophores—iron-binding proteins that solubilize Fe^3+^ into a bioavailable form. These siderophores form complexes with Fe^3+^, reducing it to Fe^2+^ for uptake by plant roots. Similarly, PSB like *Azotobacter*, *Bacillus*, and *Rhizobium* secrete enzymes and organic acids to solubilize insoluble phosphate, making it available to plants (Mitter et al., 2013).

By improving nutrient availability, biofertilizers reduce the need for chemical fertilizers, lower agricultural production costs, and contribute to environmental sustainability. Additionally, these microbes increase organic matter content in the soil, mobilize nutrients from the rhizosphere, and aid in soil fertility recovery through various direct and indirect mechanisms (Gamalero et al., 2020).

Arbuscular mycorrhizal fungi play a pivotal role in regulating plant–soil interactions, especially under elevated atmospheric CO_2_ conditions. As CO_2_ levels rise, plant photosynthesis and carbon assimilation typically increase, leading to a greater flow of carbohydrates into the rhizosphere. This enhanced carbon availability supports more robust AMF colonization, resulting in increased fungal biomass, denser hyphal networks, and greater production of glomalin, a glycoprotein crucial for soil structure and long-term carbon stability. The mutualistic relationship between plants and AMF becomes especially important under abiotic stresses like drought and salinity. AMF enhances plant tolerance by improving water and nutrient uptake, maintaining photosynthetic efficiency, and supporting osmotic balance. These physiological benefits, in turn, help sustain carbon transfer from plants to soil, even in stressful environments. The enhanced hyphal networks and glomalin secretion contribute to the formation of stable soil aggregates, which help protect soil organic matter and promote long-term carbon storage. Furthermore, elevated CO_2_ levels combined with AMF colonization foster greater microbial diversity and increase rhizodeposition, exudation of organic compounds from roots, thereby accelerating carbon cycling and nutrient turnover in the soil ecosystem. However, the effectiveness of these interactions is not uniform; it varies with plant genotype, AMF species, and the nature of the environmental stress. Still, AMF serve as key ecological buffers and facilitators, supporting both plant productivity and ecosystem-level carbon sequestration under changing climatic conditions.

Beyond AMF, a broader community of soil microorganisms, including bacteria, fungi, and archaea, plays a vital role in the biogeochemical cycling of carbon. With rising atmospheric CO_2_, traditional carbon mitigation strategies such as emission reduction and energy efficiency face scaling challenges. This has led to growing interest in biologically mediated carbon sequestration approaches, particularly those that convert CO_2_ into stable mineral forms. One promising approach is the microbial-induced precipitation of carbonate minerals, such as calcite, magnesite, and dolomite. Microorganisms like *Sporosarcina pasteurii* facilitate this process through microbial-induced calcium carbonate precipitation (MICCP), which involves the enzymatic activity of carbonic anhydrase (CA) to accelerate the conversion of CO_2_ into stable carbonates (Mukhtar et al., 2019; Dupraz et al., 2009). This not only locks carbon into mineral form but also helps reinforce geological formations by sealing fractures, improving the security of subsurface carbon storage. Cyanobacteria, such as *Microcoleus chthonoplastes*, further contribute to carbon capture by converting CO_2_ into durable calcium carbonate structures using sunlight as an energy source (Jansson and Northen, 2010; Kamennaya et al., 2012). These biomineralization processes offer innovative, nature-based solutions for long-term CO_2_ sequestration.

Altogether, integrating microbial mechanisms, especially AMF symbiosis under high CO_2_ with mineral-based carbon fixation strategies presents a promising frontier in climate mitigation. Advancing our understanding of these microbial–plant–soil interactions is essential for developing scalable, sustainable carbon management systems that align with ecological principles and global carbon reduction goals (Khan et al., 2021b; Mukhtar et al., 2019).

### 6.2 Climate change mitigation

Climate change, marked by increasing CO_2_ levels and rising temperatures, has significantly impacted ecological processes, including the interactions between plants and microbial communities (Fahad et al., 2020). The rise in CO_2_ alters root exudates by increasing carbon concentration in the root zone, which affects the composition and activity of soil microbes. This change disrupts the ratio of plant chemo attractants and the carbon-to-nitrogen (C/N) ratio, potentially altering the mutualistic relationships between plants and beneficial microbes.

#### 6.2.1 The potential of microbe-mediated mineral transformations in carbon sequestration and mitigating climate change

Microorganisms, including bacteria, fungi, and archaea, are essential for the biogeochemical cycling of elements, particularly carbon, and play a key role in long-term carbon sequestration in stable mineral forms (Khan et al., 2021b). As CO_2_ levels rise, traditional methods of reducing emissions and increasing energy efficiency have been challenging on a large scale, leading to interest in carbon sequestration through the conversion of CO_2_ into stable carbonate minerals like calcite, magnesite, and dolomite. This biomimetic approach utilizes biological catalysts, such as CA, to accelerate CO_2_ fixation into carbonate minerals (Mukhtar et al., 2019). AMF respond to high CO_2_ (eCO_2_) with enhanced root colonization, spore density, and biomass due to increased plant carbon input. Enhanced fungal activity increases nutrient acquisition (e.g., phosphorus and zinc), stimulates microbially mediated mineral weathering, and enables soil carbon sequestration via glomalin production and organic matter stabilization (Laza et al., 2023). Under stressful environments (e.g., drought and nutrient stress), AMF increase plant tolerance by enhancing water use efficiency and mobilizing bound minerals, often in combination with PSB. Benefits are, however, modulated by soil fertility, with heavy carbon investment in AMF potentially limiting plant growth when returns are low in nutrients. Understanding these processes is essential to the optimization of the potential of AMF in sustainable crop systems under a changing climate (AbdElgawad et al., 2022).

Field experiments have shown that eCO_2_ can cause extreme changes in AMF community structure. For example, long-term eCO_2_ exposure in submerged paddy soils led to the reduction of frequently occurring AMF species such as *Claroideoglomus* and *Glomus*, whereas *Scutellospora* species were dominant, reflecting their tolerance at high CO_2_ levels (Panneerselvam et al., 2020). This change is caused by variations in physiological and ecological factors affecting carbon utilization efficiency, tolerance to stress, and symbiotic compatibility with host plants. *Scutellospora* species have been reported to exhibit greater adaptation to carbon-rich environments, sustaining consistent symbiotic performance and nutrient exchange under these changed conditions. These changes in composition can impact the mobilization of mineral nutrients and the general functionality of the soil–plant system, especially in scenarios of nutrient deficiency or enhanced susceptibility to stress.

The AMF mineral interaction is controlled by abiotic factors (e.g., redox potential, soil water, and pH) and by the co-occurrence of microbial species. During low-phosphate conditions, AMF may interact with PSB to solubilize inorganic phosphate from mineral complexes, augmenting microbe–mineral synergy. Elevated CO_2_ may stimulate synergistic effects by increasing rhizospheric carbon input, and consequently, increasing associated microbial activity, driving mineral transformation. These interactions control the formation of secondary minerals and biogenic phases, contributing to soil weathering and long-term mineral cycling. MICCP by organisms like *S. pasteurii* enhances CO_2_ storage by increasing dissolved CO_2_ in subsurface water or precipitating it as carbonate minerals. This process not only stabilizes CO_2_ but also helps seal fractures in geological formations, improving long-term CO_2_ storage security (Dupraz et al., 2009). Cyanobacteria, such as *M. chthonoplastes*, also contribute to carbon capture and sequestration by converting CO_2_ into recalcitrant calcium carbonate biominerals using solar energy (Jansson and Northen, 2010; Kamennaya et al., 2012). These microbial processes offer promising solutions for mitigating climate change by securely transforming CO_2_ into stable mineral forms.

### 6.3 Sustainable agriculture

Microbial biotechnology offers tools to enhance resource use efficiency, reduce dependency on agrochemicals, and address biotic and abiotic stresses. Beneficial microbes, including PGPR and AMF, improve nutrient availability, pathogen resistance, and stress tolerance ((Kumar et al., 2025; De Bruijn, 2015). PGPR improves nutrient uptake and reduce chemical input dependency (Etesami and Adl, 2020; Olivares et al., 2013). Microbes activate systemic resistance pathways, reducing the incidence of soil-borne diseases in crops such as rice and maize. Advances in microbial biotechnology, such as genetically engineered microbial strains, offer new avenues for addressing the challenges of modern agriculture.

### 6.4 Bioremediation of contaminated soils

Microorganisms are integral to bioremediation efforts, contributing significantly to pollutant detoxification and soil health restoration (Ayangbenro and Babalola, 2017). They play a crucial role in heavy metal remediation through biosorption, facilitated by exopolysaccharides and siderophores, which immobilize toxic metals such as cadmium and lead (Saha et al., 2016; Beiyuan et al., 2017). Furthermore, hydrocarbon-degrading bacteria like *Pseudomonas* spp. degrade petroleum hydrocarbons enzymatically, aiding in soil recovery and fertility enhancement (Mustapha and Halimoon, 2015; Ullah et al., 2015). Combining PGPR with AMF has proven to amplify the effectiveness of phytoremediation strategies, offering a sustainable and eco-friendly approach to addressing soil contamination. Collectively, these microbial-driven processes are vital for detoxifying pollutants and fostering the rehabilitation of degraded ecosystems (Gupta and Diwan, 2017).

## 7 Challenges and future perspective

The intricacy of the soil ecosystem brings a significant challenge in understanding and manipulating microbial mediated mineral transformation. It is well understood that microbes and microbe associates play a vital role in mineral transport and availability, however, the mechanisms associated with how different microbes enhance the mineral uptake and how the mineral acquisition by the microbes takes place is still not understood properly. The microbial diversity is highly variable in soil and soil associated parameters such as pH, water availability, aeration and cation exchange capability are affected by the available microbes (Chen et al., 2024). Also, some microbes work synergistically with each other and can enhance mineral uptake while there are reports that some microbes are antagonists (Das et al., 2022). This might create a state of disequilibrium in the soil ecosystem, indirectly or directly affecting the growth of plants. Also, the interactions occurring at soil microbial level are not bipartite, these are multipartite interactions, studying the multipartite interactions may be conducted in controlled experiments, but is difficult to be monitored at the field levels (Chauhan et al., 2023). Ascertaining the taxonomy of microbes and assigning the functional role to a microbial community for nutrient uptake is another challenge that needs to be addressed (Castellano-Hinojosa and Strauss, 2021). Furthermore, it is now well understood that the structural complexity of xylem also has a role in nutrient uptake as mineral transport works in cognizance with the conducting tissue.

A major challenge lies in translating laboratory findings into the real-world application. With change and shifts in the environment in a geological and microbial stage, the controlled laboratory experiments which provide mechanistic insights into the microbial processes often fails to capture the full complexity of the natural environment (Chen et al., 2024). Bridging this gap requires iterative and integrative strategies: field-derived hypotheses should inform laboratory studies, while lab-based findings must be tested under realistic soil and climatic conditions (Wang D. et al., 2024). Numerical modeling and geochemical simulations can further aid in scaling these processes by reflecting the evolving nature of geological systems. Databases such as the Deep-Time Digital Earth (DDE) archive offer valuable insights into the temporal evolution of mineral compositions and can help identify biogenic signatures preserved over geological timescales (Wang D. et al., 2024).

With emerging tools and new techniques in synthetic biology and multi omics platforms, it has escalated in redefining a different approach to mineral–microbe–plant interactions. The study offers the ability to reprogram microbial strains with customized functional gene, where a specific targeted gene is modulated with respect with the stress, which helps in the enhancement of plant growth. For instance, *Bacillus megaterium* and *Serratia liquefaciens* are being applicated to improve the pH value of the soil and the mass concentration of NH_4_^+^, in soil with excessive lead and cadmium content contain urease synthesis (Fu et al., 2024). Engineered biosynthetic pathways can also produce novel chelators or exopolysaccharides that increase solubilization and immobilization of trace metals, improving their availability to plants or reducing their toxicity (Ghosh et al., 2024). Microbes have been known to form iron oxides or sulfides biogenically that alter the adsorption or absorption of other minerals. Many microbes are known to release acids, synthesize siderophores and enhance oxidation-reduction processes in the soil. This is going to have important consequences on the soil-mineral continuum. It is, therefore, important to identify the genes involved in the said processes (Chaudhary et al., 2023). Besides, identifying the genes, studying the gene expression in altered mineral regimes is also important. Assessing microbial communities in the presence of minerals to identify the active pathways involved in mineral transformation and nutrient acquisition is also important (Wang S. et al., 2024). Furthermore, it will be rewarding to use novel analytical techniques and interdisciplinary approach using various omics techniques to clearly pin-point how microbes in the soil alter nutrient uptake in plants and subsequently affect the plant growth and health (Diwan et al., 2022).

The application of advanced omics technologies like genomics, transcriptomics, proteomics, and metabolomics have given researchers the potential to understand and manipulate the field of microbe–mineral interactions, where they gain a system level perspective on the functional role and adaptive mechanisms of different microbial communities in various environmental stress. Genomic and transcriptomic technology has been applied to analyze the identification of genes and regulatory pathways for critical mineral transformation processes such as metal reduction, mineral weathering, and nutrient mobilization (Raza et al., 2024). Proteomics and metabolomics technologies are also parallelly applicated for understanding of the altercation in microbial responses by revealing in what ways the respective pathways are altered when the cellular activities behind minerals’ transformations, or when cells are impacted by abiotic stressors (limiting nutrients or toxicity due to metals) (Sandrini et al., 2022). For example, metagenomic and metatranscriptomics research on microbial assemblages in mine tailings have identified genes that confer the functional capacity for sulfate reduction, iron oxidation, and arsenic detoxification to microbial communities – which ultimately informs the design of successful bioaugmentation approaches (Li et al., 2022). Furthermore, the integration of multi-omics has led to the construction of genome-scale metabolic models (GEMs), which allow for the simulation and prediction of microbial behavior in complex mineral environments. These models not only deepen our mechanistic understanding of microbe–mineral systems but also enable the development of predictive tools to optimize biogeochemical processes for applications such as bioremediation, sustainable resource extraction, and the synthesis of novel biomaterials (Kong et al., 2025). Therefore, multi-omics-driven insights with genome-based modeling frameworks holds a significant promise for advancing mineral biotechnologies and promoting environmentally sound and efficient industrial practices.

One of the most promising research directions is the combination of genome-edited crops with synthetic microbes. With the development of CRISPR/Cas9 and associated biotechnologies, genetically engineered plants with enhanced root architecture, metal uptake efficacy, or detoxification can now be designed. Co-engineering plant and microbial systems provides a synergistic solution to increased abiotic stress tolerance, phytoremediation, and nutrient cycling (Chen et al., 2024). For example, the combination of microbes specifically designed for iron chelation or phosphate solubilization with crops designed for increased nutrient acquisition can significantly boost agricultural productivity from marginal or contaminated soils (Pattnaik et al., 2021; Mishra et al., 2023).

In summary, overcoming microbial-mediated mineral transformation challenges involves a multidisciplinary approach involving microbial ecology, soil science, systems biology, and plant physiology. Future work should focus on the development of stable synthetic consortia, dynamic simulation models, and plant–microbe co-engineering strategies to engineer sustainable and resilient agroecosystems that can withstand environmental stressors and foster long-term soil health.

## 8 Conclusion

Microbially mediated mineral transformation is essential for nutrient cycling, soil fertility, and plant growth. Microorganisms have a very strong impact on the bioavailability of vital macro- and micronutrients through dissolution, precipitation, redox conversions, and chelation, thus plant growth and stability directly. Microbe–mineral conversions also contribute a vital role in bioremediation, thus providing eco-friendly alternatives to remediate soil pollution and heavy metal toxicity. Despite substantial advances, the functional dynamics of such conversions in the complex soil ecosystem are a challenge, especially under dynamic environmental conditions.

Future research needs to focus on integration of novel approaches, such as multi-omics and synthetic biology, to define microbial functionalities on a systems level, and microbial strain development, which is specifically tailored for mineral transformation based on specific environmental or agronomic conditions. In addition, integration of laboratory-sourced research with field-sourced applications will be needed. An interdisciplinary platform focusing on geochemistry, microbial ecology, and plant physiology will be needed for optimization of novel biofertilizers and precision microbial consortia, which can contribute toward sustainable agriculture production and ecological restoration processes as we all must work together for green earth and knowledge could mechanistically guide the highly promising approaches to use microbes for more sustainable plant nutrition.
